# Analysis on the efficacy and safety of chemotherapy combined with or without bevacizumab after CRS+HIPEC in patients with malignant peritoneal mesothelioma: a single-center retrospective study

**DOI:** 10.3389/fonc.2026.1733967

**Published:** 2026-02-03

**Authors:** Zhi-Ran Yang, Xin-Li Liang, Xin-Bao Li, Xin-Jing Zhang, He-Liang Wu, Yan-Dong Su, Yan Li, Song-Lin An

**Affiliations:** 1 Department of Peritoneal Cancer Surgery, Beijing Shijitan Hospital, Capital Medical University, Beijing, China; 2 Department of Surgical Oncology, Beijing Tsinghua Changgung Hospital, Tsinghua University, Beijing, China

**Keywords:** bevacizumab, cytoreductive surgery, hyperthermic intraperitoneal chemotherapy, malignant peritoneal mesothelioma, pemetrexed/platinum-based chemotherapy, safety, survival

## Abstract

**Objective:**

To investigate the efficacy and safety of pemetrexed/platinum-based chemotherapy combined with or without bevacizumab after cytoreductive surgery (CRS) with hyperthermic intraperitoneal chemotherapy (HIPEC) in the treatment of patients with malignant peritoneal mesothelioma (MPM).

**Methods:**

A retrospective non-randomized study was performed on 205 MPM patients treated with CRS+HIPEC at our institution. A total of 97 eligible patients were analyzed: 58 patients who received postoperative chemotherapy combined with bevacizumab (C&B) and 39 patients who received chemotherapy alone (C) were divided into a study group and a control group, respectively. The patients were also divided into the bevacizumab-exposed subgroup and the bevacizumab-unexposed subgroup based on whether they had a history of bevacizumab infusion. Clinicopathological data and follow-up information were statistically analyzed. Independent prognostic factors were identified via survival analysis, and the safety of combination therapy was assessed via adverse event analysis.

**Results:**

As of the follow-up cutoff date of July 1, 2025, in both the subgroups with and without a history of bevacizumab infusion, there was no statistically significant difference between the control and study groups in baseline pathological characteristic parameters (*p* > 0.05). Survival analysis revealed that in the subgroup of patients with a history of bevacizumab infusion, the difference in median overall survival (mOS) between the control and study groups was statistically significant (31.9 months *vs.* NR; *p* = 0.031), and the difference in median disease-free survival (mDFS) between the control and study groups was statistically significant (12.5 months *vs*. NR; *p* = 0.001); in the subgroup of patients without a history of bevacizumab infusion, the difference in mOS between the control and study groups was also statistically significant (20.5 months *vs.* NR; *p* = 0.001), and the difference in mDFS between the control and study groups was statistically significant (13.2 *vs*. 36.2 months; *p* = 0.001). The Cox regression model found that postoperative C&B was an independent prognostic factor (*p* = 0.009, HR = 0.081, 95% CI: 0.012–0.526) in the subgroup with a history of bevacizumab infusion, and the Ki-67 index (*p* = 0.043, HR = 2.563, 95% CI: 1.029–6.386) and postoperative C&B were independent prognostic factors (*p* = 0.01, HR = 0.086, 95% CI: 0.032–0.232) in the subgroup with no bevacizumab treatment history. A total of 101 cases of grade 1~2 adverse events in the study group were revealed via adverse event analysis, with common events including nausea/vomiting, fatigue, leukopenia/neutropenia, thrombocytopenia, anemia, abnormal liver function, hypertension, and proteinuria. There were four cases with grade 3 adverse events, mainly leukopenia/neutropenia, hypertension, and proteinuria. In the control group, there were 84 cases of grade 1~2 adverse events, and three cases of grade 3 adverse events were observed.

**Conclusion:**

Our exploratory findings suggest that bevacizumab combined with pemetrexed/platinum-based chemotherapy may be a therapeutic option for MPM patients after CRS+HIPEC; however, a prospective, randomized controlled clinical study is needed to validate our findings.

## 1 Introduction

Malignant peritoneal mesothelioma (MPM), with the biological characteristic of diffuse and invasive growth along the peritoneal surface, is an extremely rare malignant tumor originating from peritoneal mesothelial cells (1, 2). The incidence of MPM is approximately 0.41~1.94/10^5^, accounting for approximately 15% of all mesotheliomas (3–6). Histologically, MPM is mainly classified into three subtypes: epithelioid, sarcomatoid, and biphasic. The epithelioid subtype is shown with the better prognosis, compared with the sarcomatoid and biphasic subtypes (2).

The non-specific symptoms could be found in the MPM patients, including abdominal pain, abdominal distension, fatigue, and weight loss. A study based on the Surveillance, Epidemiology, and End Results (SEER) database found that the 1-year survival rate of MPM patients is approximately 46%, with the 5-year survival rate approximately 20% (7). Commonly used chemotherapy regimens for MPM include pemetrexed, platinum-based drugs, and gemcitabine, which can prolong the median overall survival (mOS) of patients to 8.7~10.3 months (8). Cytoreductive surgery (CRS) combined with hyperthermic intraperitoneal chemotherapy (HIPEC) can further significantly extend the survival of some patients. However, due to the rarity of MPM, the misdiagnosis rate in clinical practice is relatively high, and there is a lack of a standardized treatment system. Most clinical trials and innovative drugs are mainly targeted at malignant pleural mesothelioma. Nevertheless, a growing body of studies (7, 9–11) have shown that MPM and malignant pleural mesothelioma have different immunological and molecular biological backgrounds. Therefore, it is necessary to carry out clinical trials and develop treatment regimens specifically for MPM to improve the survival rate of patients.

Pemetrexed + platinum combined with bevacizumab has currently become the standard first-line treatment regimen for malignant pleural mesothelioma (12). However, treatment protocols for MPM still draw on or follow those for malignant pleural mesothelioma. It is likely primarily caused by the rarity and poor prognosis of MPM, as some patients are already in advanced stages at diagnosis. Additionally, the great technical difficulty of CRS+HIPEC surgery means that some patients can only receive palliative treatment alone. As a specialized peritoneal cancer diagnosis and treatment center, nearly 3,000 CRS+HIPEC surgeries for peritoneal cancer were performed in our department, accumulating extensive surgical experiences, and a comprehensive clinicopathological specimen database was hereby established. Based on this foundation, the short-term efficacy and long-term prognosis of patients who received pemetrexed + platinum therapy with or without bevacizumab after CRS+HIPEC at our center were retrospectively analyzed in this study, which was intended to provide evidence for treatment regimens for MPM patients.

## 2 Materials and methods

### 2.1 Clinical data

This was a retrospective non-randomized study; 205 MPM patients had undergone CRS+HIPEC surgery at our hospital from August 2016 to February 2025, and the patients with cytoreduction (CC) scores of 2/3, with missing follow-up information, and who did not receive postoperative treatment at our center were excluded from this study. The eligible patients were divided into two groups based on whether bevacizumab was added to postoperative treatment: the study group [chemotherapy combined with bevacizumab (C&B)] and the control group [chemotherapy alone (C)].

Moreover, in order to eliminate the interference of the history of bevacizumab infusion, the patients were also divided into the bevacizumab-exposed subgroup and the bevacizumab-unexposed subgroup based on whether they had a history of bevacizumab infusion. Clinicopathological data were statistically analyzed, including clinicopathological characteristics, surgical parameters, treatment conditions, adverse events, and follow-up information. The study protocol was approved by the ethics committee of our hospital [2015- (28)], and all patients provided signed informed consent. All patients were included and excluded according to the following inclusion criteria and exclusion criteria.

Inclusion criteria: 1) pathologically confirmed MPM with complete clinicopathological data and follow-up information; 2) Eastern Cooperative Oncology Group (ECOG) performance status score ≤2, with an expected survival of more than 3 months; 3) presence of at least one measurable lesion; 4) peripheral blood leukocyte count ≥3.5 × 10^9^/L and platelet count ≥ 90 × 10^9^/L; 5) basically normal liver and kidney function; and 6) cardiopulmonary function and other major organs capable of tolerating long-duration major surgery.

Exclusion criteria: 1) preoperative examination revealing multiple metastases (e.g., to the lungs, brain, bones, or liver); 2) impaired function of major organs or abnormal coagulation function; 3) preoperative imaging suggesting obvious mesenteric contracture; 4) unhealed surgical incisions; 5) presence of severe complications (e.g., intestinal obstruction, gastrointestinal perforation, intestinal fistula) or obvious gastrointestinal bleeding; 6) resistant hypertension or abnormal proteinuria (urine protein ++ or 24-hour urine protein > 1.0 g); and 7) pregnant women, lactating women, or patients with mental illness.

### 2.2 Methods

#### 2.2.1 Preoperative laboratory examination and evaluation

Preoperative laboratory examinations were performed to evaluate tumor invasion (13), mainly including the following. 1) Serological tests: baseline complete blood count, liver and kidney function, coagulation function, myocardial enzymes, etc. 2) Serum tumor markers: carcinoembryonic antigen (CEA), alpha-fetoprotein (AFP), carbohydrate antigen 199 (CA199), carbohydrate antigen 125 (CA125), etc. 3) Imaging examinations: contrast-enhanced chest/abdominal/pelvic CT with 3D reconstruction, total gastrointestinal iodine water contrast, renal dynamic imaging, bone scan, pulmonary function test, and others. Based on the results of the aforementioned laboratory examinations, the baseline status, organ function, and tumor burden of patients were comprehensively evaluated. The included patients underwent standardized CRS+HIPEC at our hospital.

#### 2.2.2 CRS+HIPEC

In accordance with expert consensus (13), the standardized CRS+HIPEC procedure is performed by the specialized peritoneal cancer treatment team of this center, with the main steps as follows. 1) Laparotomy and comprehensive exploration of the abdominal and pelvic cavities, followed by the Peritoneal Cancer Index (PCI) score. 2) Maximal CRS based on the extent of abdominal and pelvic tumor invasion: all visible and unresectable tiny tumor nodules are ablated using argon beam coagulator or electrocautery. For cases with extensive invasion, combined resection of partial organs and peritoneum is often required, followed by completeness of the CC score. 3) Open HIPEC after CRS. The protocol is formulated as follows: 120 mg docetaxel + 120 mg cisplatin, or 120 mg cisplatin + 30 mg mitomycin C, with the solvent of 3,000 mL normal saline, is used for the patient’s circulation at a flow rate of 400 mL/min for 60 minutes after heating to 43.0 °C ± 0.5 °C. 4) After HIPEC completion, gastrointestinal reconstruction is performed, with enterostomy, if necessary. The abdomen is closed after thorough hemostasis.

#### 2.2.3 Postoperative treatment

After CRS+HIPEC, vigilance is required for severe adverse events and perioperative death. Treatment is initiated 4–6 weeks postoperatively, and patients are divided into the study group and the control group. The chemotherapy regimen consists of pemetrexed combined with platinum. In particular, patients with good economic conditions, no bleeding risk, and no history of hypertension and proteinuria were more inclined to increase the use of bevacizumab on the basis of chemotherapy. The dose of bevacizumab is 7.5 mg/kg, with a 21-day treatment cycle for a total of six cycles.

### 2.3 Efficacy evaluation

#### 2.3.1 Survival analysis

Overall survival (OS) and disease-free survival (DFS) of patients in the study were analyzed statistically. The mOS was defined as the time from CRS+HIPEC surgery to death or the end of follow-up. The median DFS (mDFS) was defined as the time from CRS+HIPEC surgery to disease progression or the end of follow-up.

#### 2.3.2 Study indicators

The study indicators mainly included the following three categories. 1) Clinicopathological characteristics: gender, age, ECOG performance status, previous treatment history, previous surgical history, histopathological type, postoperative pathological findings, preoperative CA125 level, etc. 2) Surgery-related information: PCI, status of partial organ/regional peritoneal resection, HIPEC regimen, number of anastomoses, etc. 3) Survival status: survival status and OS.

### 2.4 Adverse events

Serious adverse events (SAEs) and death-related events in patients of both groups were statistically analyzed. Adverse events were evaluated and graded in accordance with the Common Terminology Criteria for Adverse Events (CTCAE) developed by the National Cancer Institute (NCI). All adverse events occurring from the start of treatment to 1 month after the end of treatment or the end of follow-up were statistically analyzed.

### 2.5 Follow-up

The follow-up content included the survival status, general condition, grading of adverse events, and laboratory tests. The follow-up methods included inpatient/outpatient revisit records or telephone follow-up. The last follow-up date was July 1, 2025.

### 2.6 Statistical analysis

Data analysis was performed using Microsoft Excel 2016 and the SPSS 27.0 statistical software. Quantitative data were analyzed using the t-test or the rank-sum test. Categorical data were analyzed using the chi-square test or Fisher’s exact test. Survival analysis was performed using the Kaplan–Meier survival function and log-rank test; the independent prognostic factors affecting survival were identified using the Cox proportional hazards regression model. *p* < 0.05 was considered statistically significant.

## 3 Results

### 3.1 Baseline clinicopathological characteristics in this study

**Figure 1:**
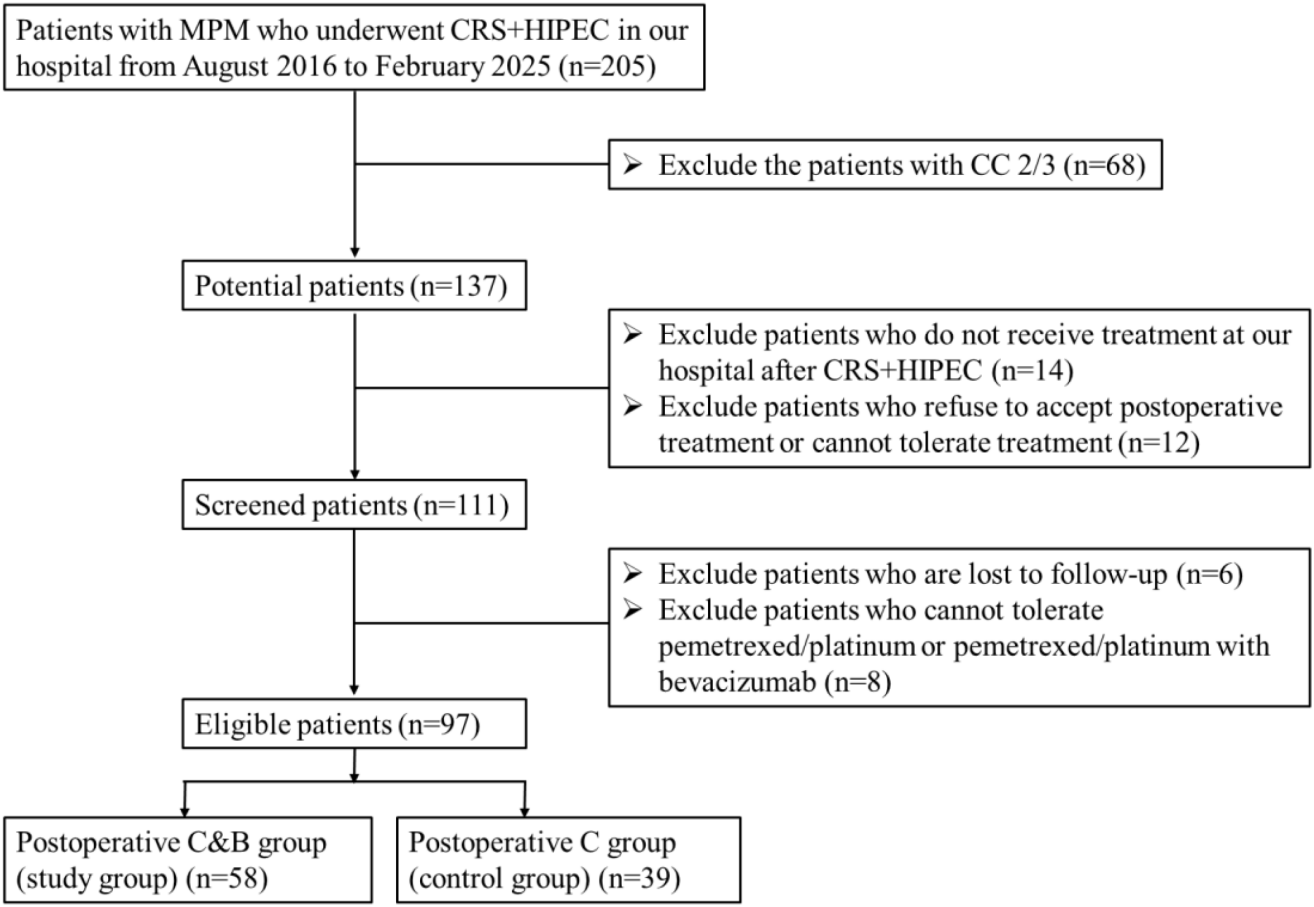
Flowchart of patient inclusion. MPM, malignant peritoneal mesothelioma; CRS+HIPEC, cytoreductive surgery with hyperthermic intraperitoneal chemotherapy; CC, completeness of cytoreduction; Postoperative C&B, postoperative chemotherapy combined with bevacizumab; Postoperative C, postoperative chemotherapy.

The research was a retrospective non-randomized study of a total of 205 patients with MPM who underwent CRS+HIPEC surgery in our hospital from August 2016 to February 2025. The patients were screened according to the exclusion criteria shown in **Figure 1**, and 97 patients were included in this retrospective study, in which 58 patients who received postoperative chemotherapy combined with bevacizumab (postoperative C&B) and 39 patients who received postoperative chemotherapy (postoperative C) alone were divided into the study group and the control group, respectively.

The 97 enrolled MPM patients were divided into groups according to whether there was a history of bevacizumab infusion. There were 29 patients with a history of bevacizumab infusion, including 10 in the control group and 19 in the study group; there were 68 patients without a history of bevacizumab infusion, including 29 in the control group and 39 in the study group. The baseline clinicopathological characteristics of patients are shown in **Table 1**, and there was no statistically significant difference (*p* > 0.05).

**Table 1:** Baseline clinical pathological characteristics of the groups.

Characteristics n (%)	With history of bevacizumab infusion (n = 29)		*p*	Without history of bevacizumab infusion (n = 68)		*p*
	CG (n = 10)	SG (n = 19)		CG (n = 29)	SG (n = 39)	
Sex			0.089			0.193
*Male*	3 (30.0)	12 (63.2)		11 (37.9)	21 (53.8)	
*Female*	7 (70.0)	7 (36.8)		18 (62.1)	18 (46.2)	
Age			0.331			0.142
*≤56*	5 (50.0)	13 (68.4)		15 (51.7)	27 (69.2)	
*>56*	5 (50.0)	6 (31.6)		14 (48.3)	12 (30.8)	
ECOG			0.547			0.458
*0*	5 (50.0)	12 (63.1)		16 (55.2)	26 (66.7)	
*1*	4 (40.0)	4 (21.1)		7 (24.1)	5 (12.8)	
*2*	1 (10.0)	3 (15.8)		6 (20.7)	8 (20.5)	
Surgery history			0.976			0.649
*No*	8 (80.0)	13 (68.4)		15 (51.7)	18 (46.2)	
*Yes*	2 (20.0)	6 (31.6)		14 (48.3)	21 (53.8)	
With history of chemotherapy			0.089			0.645
*No*	7 (70.0)	7 (36.8)		17 (58.6)	25 (64.1)	
*Yes*	3 (30.0)	12 (63.2)		12 (41.4)	14 (35.9)	
Histotype			0.775			0.45
*Epithelioid*	8 (80.0)	16 (84.2)		27 (93.1)	35 (89.7)	
*Non-epithelioid*	2 (20.0)	3 (15.8)		2 (6.9)	4 (10.3)	
HIPEC regimen			0.896			0.278
*Docetaxel + cisplatin*	6 (60.0)	10 (52.6)		15 (51.7)	27 (69.2)	
*Cisplatin + mitomycin C*	4 (40.0)	9 (47.4)		14 (48.3)	12 (30.8)	
Vascular tumor embolism			0.482			0.855
*No*	8 (80.0)	17 (89.5)		25 (86.2)	33 (84.6)	
*Yes*	2 (20.0)	2 (10.5)		4 (13.8)	6 (15.4)	
Lymph node metastasis			0.076			0.41
*No*	4 (40.0)	14 (73.7)		22 (75.9)	26 (66.7)	
*Yes*	6 (60.0)	5 (26.3)		7 (24.1)	13 (33.3)	
CA125 elevation			0.523			0.754
*No*	7 (70.0)	11 (57.9)		16 (55.2)	23 (58.9)	
*Yes*	3 (30.0)	8 (42.1)		13 (44.8)	16 (41.1)	
Ki-67 index			0.868			0.208
*≤9%*	6 (60.0)	12 (63.2)		9 (31.0)	18 (46.1)	
*>9%*	4 (40.0)	7 (36.8)		20 (69.0)	21 (53.9)	
PCI			0.051			0.855
*≤20*	3 (30.0)	13 (68.4)		17 (58.6)	22 (56.4)	
*>20*	7 (70.0)	6 (31.6)		12 (41.4)	17 (43.6)	
Organs removed			0.359			0.381
*≤2*	4 (40.0)	11 (57.9)		15 (51.7)	16 (41.0)	
*>2*	6 (60.0)	8 (42.1)		14 (48.3)	23 (59.0)	
Peritoneal resection			0.518			0.964
*≤5*	6 (60.0)	9 (47.4)		11 (37.9)	15 (38.5)	
*>5*	4 (40.0)	10 (52.6)		18 (62.1)	24 (61.5)	
Anastomosis number			0.648			0.608
*0*	4 (40.0)	11 (57.9)		13 (44.8)	18 (46.1)	
*1*	4 (40.0)	5 (26.3)		13 (44.8)	14 (35.9)	
*2*	2 (20.0)	3 (15.8)		3 (10.4)	7 (18.0%)	

CG, control group; SG, study group; ECOG, Eastern Cooperative Oncology Group; PCI, peritoneal cancer index; HIPEC, hyperthermic intraperitoneal chemotherapy; CA125, carbohydrate antigen 125.

### 3.2 Analysis of survival and prognostic factors in the study

#### 3.2.1 The survival analysis

**Figure 2:**
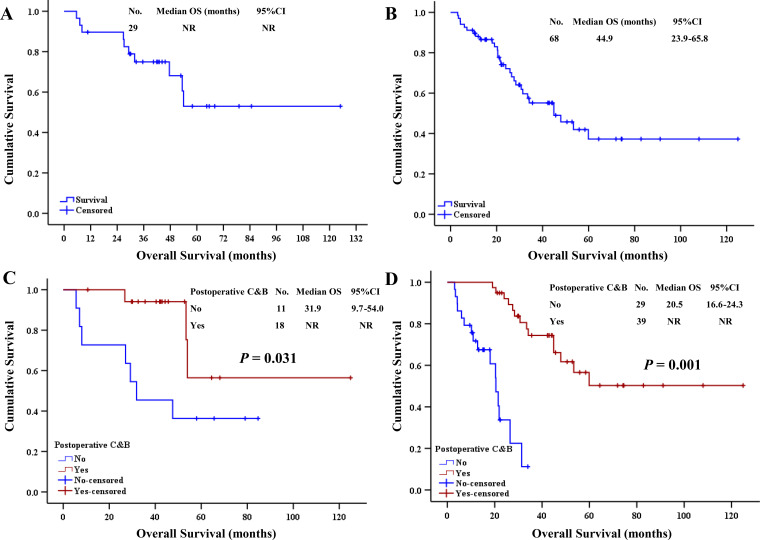
The survival analysis of the groups. **(A)** Overall survival analysis of subgroup with history of bevacizumab infusion. **(B)** Overall survival analysis of subgroup without history of bevacizumab infusion. **(C)** Survival curve analysis of control and treatment groups in subgroup with history of bevacizumab infusion. **(D)** Survival curve analysis of control and treatment groups without history of bevacizumab infusion. NR, not reached; Postoperative C&B, postoperative chemotherapy combined with bevacizumab.

As of July 1, 2025, in the subgroup of patients with a history of bevacizumab infusion, the mOS was not reached (95% CI: not reached) (**Figure 2A**), with 19 (65.5%) patients alive and 10 (34.5%) deceased; the mDFS was 36.2 months (95% CI: 18.0–54.3).

Among the subgroup of patients without a history of bevacizumab infusion, the mOS was 44.9 months (95% CI: 23.9–65.8) (**Figure 2B**), with 38 (55.9%) patients alive and 30 (44.1%) deceased; the mDFS was 26.0 months (95% CI: 20.4–31.5).

In the subgroup of patients with a history of bevacizumab infusion, the difference in mOS between the control and study groups was statistically significant (31.9 months *vs*. NR; *p* = 0.031) (**Figure 2C**); the difference in mDFS between the control and study groups was statistically significant (12.5 months *vs*. NR; *p* = 0.001).

In the subgroup of patients without a history of bevacizumab infusion, the difference in mOS between the control and study groups was also statistically significant (20.5 months *vs.* NR; *p* = 0.001) (**Figure 2D**); the difference in mDFS between the control and study groups was statistically significant (13.2 *vs*. 36.2 months; *p* = 0.001).

#### 3.2.2 Univariate analysis

**Figure 3:**
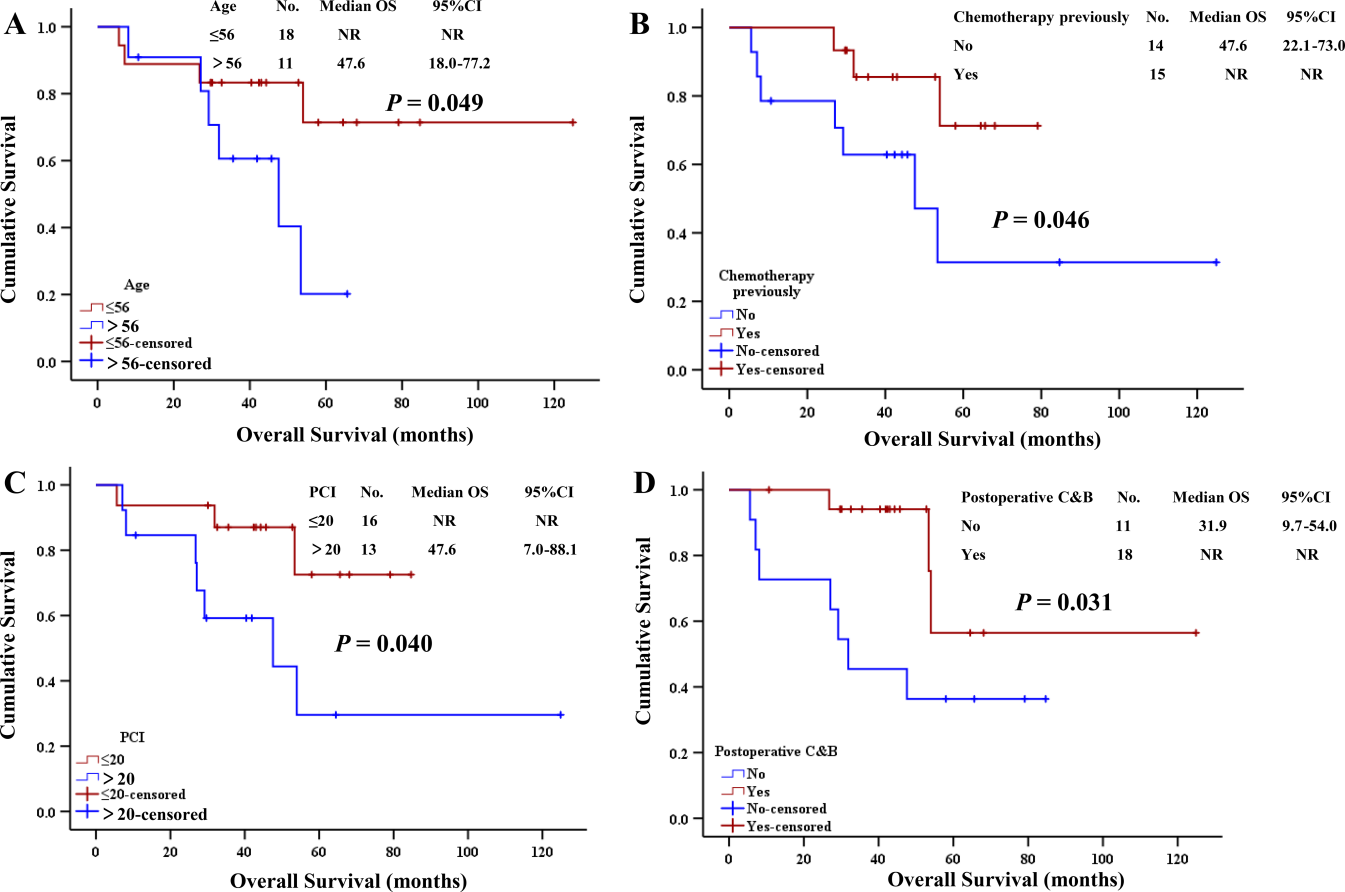
Univariate analysis of survival in the group with history of bevacizumab infusion. **(A)** Age. **(B)** With history of chemotherapy previously. **(C)** PCI. **(D)** Postoperative C&B. NR, not reached; C&B, chemotherapy combined with bevacizumab; PCI, peritoneal cancer index.

Univariate analysis revealed the following factors influencing the prognosis of MPM patients with a history of bevacizumab infusions (**Table 2**): age (**Figure 3A**) (*p* = 0.049), a history of intravenous chemotherapy (**Figure 3B**) (*p* = 0.046), PCI (**Figure 3C**) (*p* = 0.040), and postoperative C&B (**Figure 3D**) (*p* = 0.031).

**Table 2:** Univariate analysis of prognostic factors in the group with history of bevacizumab infusion.

Characteristics	N (%)	mOS (months, 95% *CI*)	χ^2^ value	*p*-Value
**Age**			3.622	**0.049**
*≤56*	18 (62.1)	NR (NR)		
*>56*	11 (37.9)	47.6 (18.0–77.2)		
Sex			1.068	0.301
*Male*	15 (51.7)	54.0 (NR)		
*Female*	14 (48.3)	NR (NR)		
ECOG *0*	17 (58.6)	NR (NR)	1.994	0.369
*1*	8 (27.6)	47.6 (20.2–74.9)		
*2*	4 (13.8)	NR (NR)		
History of surgery			0.808	0.369
*No*	21 (72.4)	53.4 (NR)		
*Yes*	8 (27.6)	NR (NR)		
**With history of chemotherapy**			3.972	**0.046**
*No*	14 (48.3)	47.6 (22.1–73.0)		
*Yes*	15 (51.7)	NR (NR)		
Histotype			2.987	0.084
*Epithelioid*	24 (82.7)	NR		
*Non-epithelioid*	5 (17.3)	53.4 (NR)		
HIPEC regimen			1.634	0.873
*Docetaxel + cisplatin*	16 (55.1)	50.2 (17.8–68.2)		
*Cisplatin + mitomycin C*	13 (44.9)	49.3 (16.2–58.9)		
Vascular tumor embolism			0.338	0.561
*No*	25 (86.2)	54.0 (NR)		
*Yes*	4 (13.8)	NR (NR)		
lymph node metastasis		N	2.012	0.156
*No*	18 (62.1)	R (NR)		
*Yes*	11 (37.9)	47.6 (13.1–82.0)		
**PCI**			4.232	**0.040**
*≤20*	16 (55.2)	NR (NR)		
*>20*	13 (44.8)	47.6 (7.0–88.1)		
CA125 elevation			0.010	0.920
*No*	18 (62.1)	NR (NR)		
*Yes*	11 (37.9)	54.0 (41.7–66.2)		
Ki-67 index			1.253	0.263
*≤9%*	11 (37.9)	NR (NR)		
*>9%*	18 (62.1)	NR (NR)		
Organs removed			1.732	0.188
*≤2*	15 (51.7)	NR (NR)		
*>2*	14 (48.3)	53.4 (26.3–80.4)		
Peritoneal resection			0.515	0.473
*≤5*	15 (51.7)	NR (NR)	1.258	0.533
*>5*	14 (48.3)	54.0 (46.0–61.9)		
Number of anastomoses				
*0*	15 (51.7)	NR (NR)		
*1*	9 (31.0)	54.0 (17.7–90.2)		
*2*	5 (17.3)	NR (NR)		
**Postoperative C&B**			4.672	**0.031**
*No*	11 (37.9)	31.9 (9.7–54.0)		
*Yes*	18 (62.1)	NR (NR)		

OS, median overall survival; NR, not reached; ECOG, Eastern Cooperative Oncology Group; PCI, peritoneal cancer index; C&B, chemotherapy combined with bevacizumab; HIPEC, hyperthermic intraperitoneal chemotherapy; CA125, carbohydrate antigen 125. The bold values means P<0.05.

**Figure 4:**
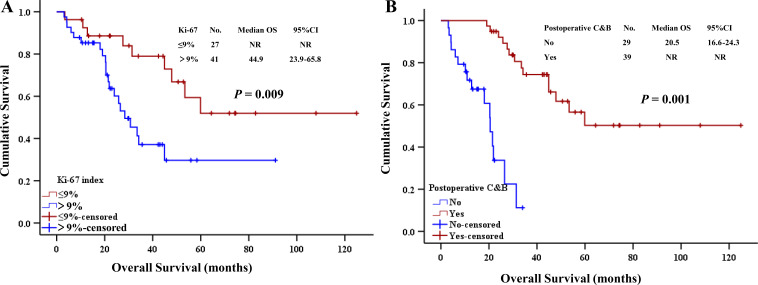
Univariate analysis of survival in the group without history of bevacizumab infusion. **(A)** Ki-67 index. **(B)** Postoperative C&B. C&B, chemotherapy combined with bevacizumab.

The factors influencing the prognosis of patients with no history of bevacizumab infusions were as follows (**Table 3**): age (*p* = 0.030), Ki-67 index (**Figure 4A**) (*p* = 0.009), and postoperative C&B (**Figure 4B**) (*p* = 0.001).

**Table 3:** Univariate analysis of prognostic factors in the group without history of bevacizumab infusion.

Characteristics	N (%)	mOS (months, 95% *CI*)	χ^2^ value	*p*-Value
**Age**			4.706	**0.030**
*≤56*	42 (61.8)	NR (NR)		
*>56*	26 (38.2)	28.4 (22.9–33.8)		
Sex			0.000	0.983
*Male*	32 (47.0)	44.9 (29.5–60.2)		
*Female*	36 (53.0)	59.9 (5.1–114.6)		
ECOG			2.058	0.357
*0*	42 (61.8)	47.9 (26.3–69.4)		
*1*	12 (17.6)	25.8 (18.3–33.2)		
*2*	14 (20.6)	NR (NR)		
History of surgery			1.171	0.279
*No*	33 (48.5)	33.5 (15.9–51.0)		
*Yes*	35 (51.5)	53.4 (39.7–67.0)		
With history of chemotherapy			0.804	0.370
*No*	42 (61.8)	53.4 (19.1–87.6)		
*Yes*	26 (38.2)	8.0 (18.4–49.9)		
Histotype			3.239	0.072
*Epithelioid*	62 (91.2)	12.9 (22.4–73.3)		
*Non-epithelioid*	6 (8.8)	4.9 (14.3–33.6)		
Vascular tumor embolism			0.196	0.658
*No*	58 (85.3)	47.9 (23.2–72.5)		
*Yes*	10 (14.7)	18.4 (8.8–80.9)		
Lymph node metastasis			0.064	0.800
*No*	43 (63.2)	NR (NR)		
*Yes*	25 (36.8)	44.9 (21.1–68.6)		
PCI			1.788	0.181
*≤20*	39 (57.3)	53.4 (38.5–68.2)		
*>20*	29 (42.7)	33.5 (20.2–46.7)		
CA125 elevation			0.062	0.804
*No*	39 (57.3)	47.9 (20.5–75.2)		
*Yes*	29 (42.7)	8.0 (29.0–60.7)		
**Ki-67 index**			6.814	**0.009**
*≤9%*	27 (39.7)	NR (NR)		
*>9%*	41 (60.3)	44.9 (23.9–65.8)		
Organs removed			0.026	0.871
*≤2*	31 (45.6)	44.9 (27.1–62.6)		
*>2*	37 (54.4)	53.4 (17.2–89.5)		
Peritoneal resection			1.092	0.296
*≤5*	26 (38.2)	34.2 (12.7–55.6)		
*>5*	42 (61.8)	13.9 (26.1–80.6)		
Anastomosis number			0.029	0.985
*0*	31 (45.6)	53.4 (15.8–90.9)		
*1*	27 (39.7)	44.9 (30.4–59.3)		
*2*	10 (14.7)	22.6 (3.4–92.3)		
**Postoperative C&B**			32.194	**0.001**
*No*	29 (42.6)	20.5 (16.6–24.3)		
*Yes*	39 (57.4)	NR (NR)		

mOS, median overall survival; NR, not reached; ECOG, Eastern Cooperative Oncology Group; PCI, peritoneal cancer index; C&B, chemotherapy combined with bevacizumab; CA125, carbohydrate antigen 125. The bold values means P<0.05.

#### 3.2.3 Multivariate analysis

Factors with *p* < 0.05 in the univariate survival analysis of the subgroup with a history of bevacizumab infusion were included in the Cox regression model for multivariate analysis (**Table 4**), and it was found that postoperative C&B was an independent prognostic factor (*p* = 0.009, HR = 0.081, 95% CI: 0.012–0.526).

**Table 4:** Multivariate survival analysis in group with history of bevacizumab infusion.

Characteristics	*Wald*	*p*	*HR*	95% *CI*
Age	1.274	0.259	2.560	0.501~13.094
History of surgery	0.606	0.436	0.524	0.103~2.664
With history of chemotherapy	0.094	0.759	0.783	0.164–3.739
PCI	1.847	0.174	3.540	0.572–21.910
**Postoperative C&B**	6.923	**0.009**	0.081	0.012–0.526

C&B, chemotherapy combined with bevacizumab. The bold values means P<0.05.

Univariate survival analysis of the subgroup with no bevacizumab treatment history included factors with *p* < 0.05 in the Cox regression model multivariate analysis (**Table 5**), and it was found that the Ki-67 index (*p* = 0.043, HR = 2.563, 95% CI: 1.029–6.386) and postoperative C&B were independent prognostic factors (*p* = 0.01, HR = 0.086, 95% CI: 0.032–0.232).

**Table 5:** Multivariate survival analysis in group without history of bevacizumab infusion.

Characteristics	*Wald*	*p*	*HR*	95% *CI*
Age	1.286	0.257	1.581	0.716–3.489
**Ki-67**	4.083	**0.043**	2.563	1.029–6.386
**Postoperative C&B**	23.407	**0.001**	0.086	0.032–0.232

C&B, chemotherapy combined with bevacizumab. The bold values means P<0.05.

### 3.3 Adverse event analysis

Adverse events in the study and control groups were analyzed separately; no grade 4 adverse events occurred in either group. Among them, grade 1~2 adverse events in the study group included the following: 16 cases of nausea/vomiting, 10 cases of fatigue, five cases of decreased appetite, 26 cases of leukopenia/neutropenia, 15 cases of thrombocytopenia, six cases of anemia, 10 cases of abnormal liver function, two cases of abnormal renal function, two cases of diarrhea/abdominal pain, two cases of intestinal obstruction, three cases of oral ulcers/stomatitis, one case of fever, two cases of pleural effusion/infection, two cases of peritoneal effusion/infection, six cases of hypertension, five cases of proteinuria, two cases of hand–foot syndrome, one case of joint pain, and one case of urinary tract infection. A total of 101 cases of grade 1~2 adverse events and four cases of grade 3 adverse events were concluded in the treatment group, compared with 84 cases of grade 1–2 adverse events and three cases of grade 3 adverse events in the control group. The incidence of adverse events was similar between the two groups, as shown in **Table 6**.

**Table 6:** Analysis of adverse events in study and control groups.

Adverse events	Study group (n = 58)			Control group (n = 39)		
	Grade 1–2	Grade 3	Grade 4	Grade 1–2	Grade 3	Grade 4
Nausea/vomiting	16	0	0	13	0	0
Fatigue	10	0	0	7	0	0
Decreased appetite	5	0	0	3	0	0
Leukopenia/neutropenia	26	1	0	22	1	0
Thrombocytopenia	15	0	0	12	0	0
Anemia	6	0	0	4	0	0
Liver function abnormality	10	0	0	7	1	0
Renal function abnormality	2	0	0	1	0	0
Bleeding	0	0	0	0	0	0
Deep vein thrombosis	0	0	0	0	0	0
Diarrhea/abdominal pain	2	0	0	1	0	0
bowel perforation	0	0	0	0	0	0
Intestinal obstruction	2	0	0	1	0	0
Oral ulcer/oral inflammation	3	0	0	1	0	0
fever	1	0	0	2	0	0
Pleural effusion/infection	2	0	0	1	0	0
Abdominal ascites/infection	2	0	0	1	0	0
Hypertension	6	2	0	3	0	0
Proteinuria	5	1	0	3	1	0
Hand–foot syndrome	2	0	0	2	0	0
Joint pain	1	0	0	0	0	0
Urinary tract infection	1	0	0	0	0	0
Total	101	4	0	84	3	0

## 4 Discussion

MPM is a relatively rare malignant tumor with non-specific symptoms, and most cases are at an advanced stage when diagnosed, which limits the recruitment for basic experimental research and clinical trials (9, 14). Therefore, currently, in clinical practice, the data on clinical treatment regimens for MPM usually come from single-center, retrospective studies with insufficient evidence-based medical evidence, or are inferred directly from research data on malignant pleural mesothelioma. In recent years, with the deepening understanding of tumors and the upgrading of innovative drugs and treatment modalities, including molecular targeted therapy and immunotherapy, revolutionary changes have taken place in the treatment of various tumors, significantly improving patients’ prognosis and quality of life. In the field of malignant mesothelioma research, most studies have focused on malignant pleural mesothelioma. However, our diagnosis and treatment center is committed to the research of peritoneal cancer, mainly including preoperative assessment of peritoneal cancer, screening of eligible patients for surgery, promotion of CRS+HIPEC surgical techniques, perioperative patient management, comprehensive treatment of postoperative and recurrent/progressive patients, and establishment of prognostic models. The basic studies and clinicopathological data statistical analysis were conducted in our comprehensive clinical patient database and specimen bank.

This study retrospectively analyzed data on the survival, prognosis, and adverse events of chemotherapy with or without bevacizumab in patients with MPM after CRS+HIPEC. The results showed that chemotherapy combined with bevacizumab may be a therapeutic option for MPM patients after CRS+HIPEC with controllable adverse reaction, primarily manifested in the following aspects: 1) in the subgroup of patients with a history of bevacizumab infusion, the difference in mOS between the control and study groups was statistically significant (31.9 months *vs.* NR; *p* = 0.031); in the subgroup of patients without a history of bevacizumab infusion, the difference in mOS between the control and study groups was also statistically significant (20.5 months *vs.* NR; *p* = 0.001). 2) The Cox regression model found that postoperative C&B was an independent prognostic factor (*p* = 0.009, HR = 0.081, 95% CI: 0.012–0.526) in the subgroup with a history of bevacizumab infusion, and the Ki-67 index (*p* = 0.043, HR = 2.563, 95% CI: 1.029–6.386) and postoperative C&B were independent prognostic factors (*p* = 0.01, HR = 0.086, 95% CI: 0.032–0.232) in the subgroup with no bevacizumab treatment history. 3) A total of 101 cases of grade 1~2 adverse events in the study group were revealed via adverse event analysis, with common events including nausea/vomiting, fatigue, leukopenia/neutropenia, thrombocytopenia, anemia, abnormal liver function, hypertension, and proteinuria; there were four cases with grade 3 adverse events, mainly leukopenia/neutropenia, hypertension, and proteinuria. In the control group, there were 84 cases of grade 1~2 adverse events, and three cases of grade 3 adverse events were observed.

In a phase II clinical trial (15), it was found that in the treatment of malignant pleural mesothelioma with pemetrexed + platinum combined with bevacizumab, 34% of patients achieved partial response (PR) and 58% had stable disease (SD). In a large-scale phase III clinical trial for malignant pleural mesothelioma (16), it was shown that compared with cisplatin/pemetrexed alone, the addition of bevacizumab to cisplatin/pemetrexed significantly prolonged PFS (9.2 months) and OS (18.8 months), whereas the PFS and OS in the cisplatin/pemetrexed alone group were 7.3 and 16.1 months, respectively.

Research related to MPM is relatively scarce. Tomonobu Koizumi (17) et al. reported a case series, pointing out that two MPM patients achieved a successful response after first-line treatment combined with bevacizumab and pemetrexed/platinum. This study further indicates that for MPM patients receiving pemetrexed + platinum combined with bevacizumab after CRS+HIPEC, the prognosis of the patient may be further improved. In the subgroup of patients with a history of bevacizumab infusion, the mOS of the control was 31.9 months, and the study group was not reached (*p* = 0.031). In the subgroup of patients without a history of bevacizumab infusion, the mOS of the control was 20.5 months, and the study group was not reached (*p* = 0.001).

In studies on MPM prognostic factors, these lines of evidence (18–23) suggested that patient age, extent of cytoreduction, histopathological type, PCI, thrombocytosis, high Ki-67 level, lymph node metastasis, and lymph node and vascular invasion were all associated with patient prognosis. Patients with old age, achievement of CC 2/3, non-epithelioid type, high PCI, high Ki-67 level, lymph node metastasis, and vascular invasion were shown with the poorer prognosis. The basic clinical pathological factors were also included in our study. Univariate analysis revealed the following factors influencing the prognosis of patients with a history of bevacizumab infusions: age, history of intravenous chemotherapy, PCI, and postoperative. The factors influencing the prognosis of patients with no history of bevacizumab infusions were as follows: age, Ki-67 index, and postoperative C&B. However, the univariate survival analysis found that in the subgroup with a history of bevacizumab infusion, postoperative C&B was an independent prognostic factor; in the subgroup with no bevacizumab treatment history, the Ki-67 index and postoperative C&B were independent prognostic factors. Based on the previous literature of our research team (2, 24, 25), this study also used a cutoff value of Ki-67 > 9%, but this cutoff may not be generalizable.

However, the research was a single-center, retrospective, non-randomized study. The evidence had some boundedness and limited extrapolation; thus, large-scale, multi-center, prospective cohort studies are urgently needed.

## Data Availability

The original contributions presented in the study are included in the article/supplementary material. Further inquiries can be directed to the corresponding authors.
